# Branch water uptake and redistribution in two conifers at the alpine treeline

**DOI:** 10.1038/s41598-021-00436-x

**Published:** 2021-11-19

**Authors:** Adriano Losso, Andreas Bär, Lucrezia Unterholzner, Michael Bahn, Stefan Mayr

**Affiliations:** 1 Department of Botany, University of Innsbruck, Sternwartestraße 15, 6020 Innsbruck, Austria; 2 Hawkesbury Institute for the Environment, Western Sydney University, Richmond, NSW 2753 Australia; 3 Department TeSAF, Università Degli Studi Di Padova, Legnaro, PD Italy; 4 Department of Ecology, University of Innsbruck, Sternwartestraße 15, 6020 Innsbruck, Austria

**Keywords:** Natural variation in plants, Plant ecology, Plant physiology

## Abstract

During winter, conifers at the alpine treeline suffer dramatic losses of hydraulic conductivity, which are successfully recovered during late winter. Previous studies indicated branch water uptake to support hydraulic recovery. We analyzed water absorption and redistribution in *Picea abies* and *Larix decidua* growing at the treeline by in situ exposure of branches to δ^2^H-labelled water. Both species suffered high winter embolism rates (> 40–60% loss of conductivity) and recovered in late winter (< 20%). Isotopic analysis showed water to be absorbed over branches and redistributed within the crown during late winter. Labelled water was redistributed over 425 ± 5 cm within the axes system and shifted to the trunk, lower and higher branches (tree height 330 ± 40 cm). This demonstrated relevant branch water uptake and re-distribution in treeline conifers. The extent of water absorption and re-distribution was species-specific, with *L. decidua* showing higher rates. *In natura*, melting snow might be the prime source for absorbed and redistributed water, enabling embolism repair and restoration of water reservoirs prior to the vegetation period. Pronounced water uptake in the deciduous *L. decidua* indicated bark to participate in the process of water absorption.

## Introduction

Trees at the treeline are exposed to contrasting conditions in summer and winter, with serious limitations in hydraulics during winter months. Accordingly, dramatic losses of hydraulic conductivity were reported in treeline conifers growing at high elevation in the European Alps^1–6^. These were caused by xylem embolism, which result from long-term exposure to frost drought and freeze–thaw stress^2,5–9^. Frost drought is induced by ice formation in soil, roots, and stems, which prevents water uptake and transport and can last for months at the treeline. Frost drought is especially relevant for evergreen conifers as radiational heating can raise needle temperatures far above that of the air, thereby increasing transpirational forces^1,10^. Consequently, water potentials (Ψ) decrease and, when critical thresholds are reached, the formation of embolism via air-seeding^11^ is induced. Freeze–thaw events, which have been reported to occur frequently in the crown of treeline conifers during winter^2,12,13^, induce embolism by bubble expansion during formation and/or melting of ice in the xylem^9,14–16^.

Previous studies^17–19^ suggested that Ψ inducing more than 50% loss of conductivity is lethal for conifer species. Though, it has been demonstrated that conifers growing at the alpine treeline can suffer even up to 100% loss of conductivity and recover during springtime^3–6^. Xylem recovery is likely based on active refilling processes^1,3,5,6,8,20^ and notably starts in late winter and thus at a time, when trees still have no access to soil water. There are some indications that treeline conifers may be able to take up water over the branches^3,21^. Foliar^20,22,23^ and bark^24,25^ water uptake have been observed in a wide range of conifer species. In particular, conifers growing in drought-prone environments have been reported to absorb water from dew^26^ and fog ^23,27,28^, improving shoot and needle water status. Under water stress conditions, fog contributed for up to 40% of the total foliar water content of *Sequoia sempervirens*^29^ and *Drimys brasiliensis*^28^. Based on deuterium (δ^2^H) labelling experiments, Cassana et al. (2016) demonstrated on *Araucaria angustifolia* that fog water cannot only be absorbed by the leaves, but also transported through the xylem to the roots and even to the soil. Recent studies^30–32^﻿ also reported reversed water flows from leaves to roots and soil, which favored hydraulic redistribution as well as growth. However, knowledge on whether and across which distances water can be redistributed within the crown to maintain a balanced water status and/or support hydraulic recovery is still lacking. This is particularly true for treeline conifers, where the repair of winter embolism is crucial for the reactivation of physiological activities during late winter. As supposed by previous studies^3,6^, trees may take advantage of melting snow for local embolism repair, but a shift of water from distal to basal branch sections or even to the trunk was not yet demonstrated. Under winter conditions, treeline conifers would benefit from long-distance water transport, as it may enable embolism repair in tree parts which do not have access to external water (e.g., the main stem is hardly covered by snow) and/or exhibit low water potential and embolism^4^. In the present study, we mimicked snow melting on the crown of specimens of the evergreen species *Picea abies* and the deciduous species *Larix decidua* growing at the alpine treeline by in situ exposure of branches to δ^2^H-labelled water. The resulting water isotopic composition of samples collected at different positions within the crown was monitored over the end of winter until beginning of summer 2017. As previous studies reported branch water uptake only for *P. abies*, we hypothesized (i) that both conifers under study can take up relevant amounts of water via the branches, (ii) that this water is redistributed within the crown depending on temperatures and ice blockages within the axes system, and (iii) that redistributed water supports xylem recovery both at the absorption site as well as other parts of the crown. Analyses were complemented by meteorological data and hydraulics measurements (i.e., Ψ and percent loss of conductivity), which enabled to monitor plant water status.

## Results

### Temperatures and precipitation

Meteorological data (Fig. 2) showed a cold period with subzero temperatures from mid-February to mid-March 2017. Daily mean temperatures increased at the end of March and remained above zero (ca. 5 °C) until mid-April, before they decreased again until the beginning of May. From February to May, snow precipitation (Fig. 2a) mainly occurred during cold periods at the beginning of March, and around mid-April and end of April 2017. From the beginning of May, temperatures started to progressively raise and snow on the soil started to melt. Mean temperatures inside the bags were slightly higher than those measured outside (ΔT: *L. decidua* 1.66 ± 0.15 °C, *P. abies* 1.06 ± 0.14 °C; data not shown).

### Isotope analyses: seasonal course

**Figure 1:**
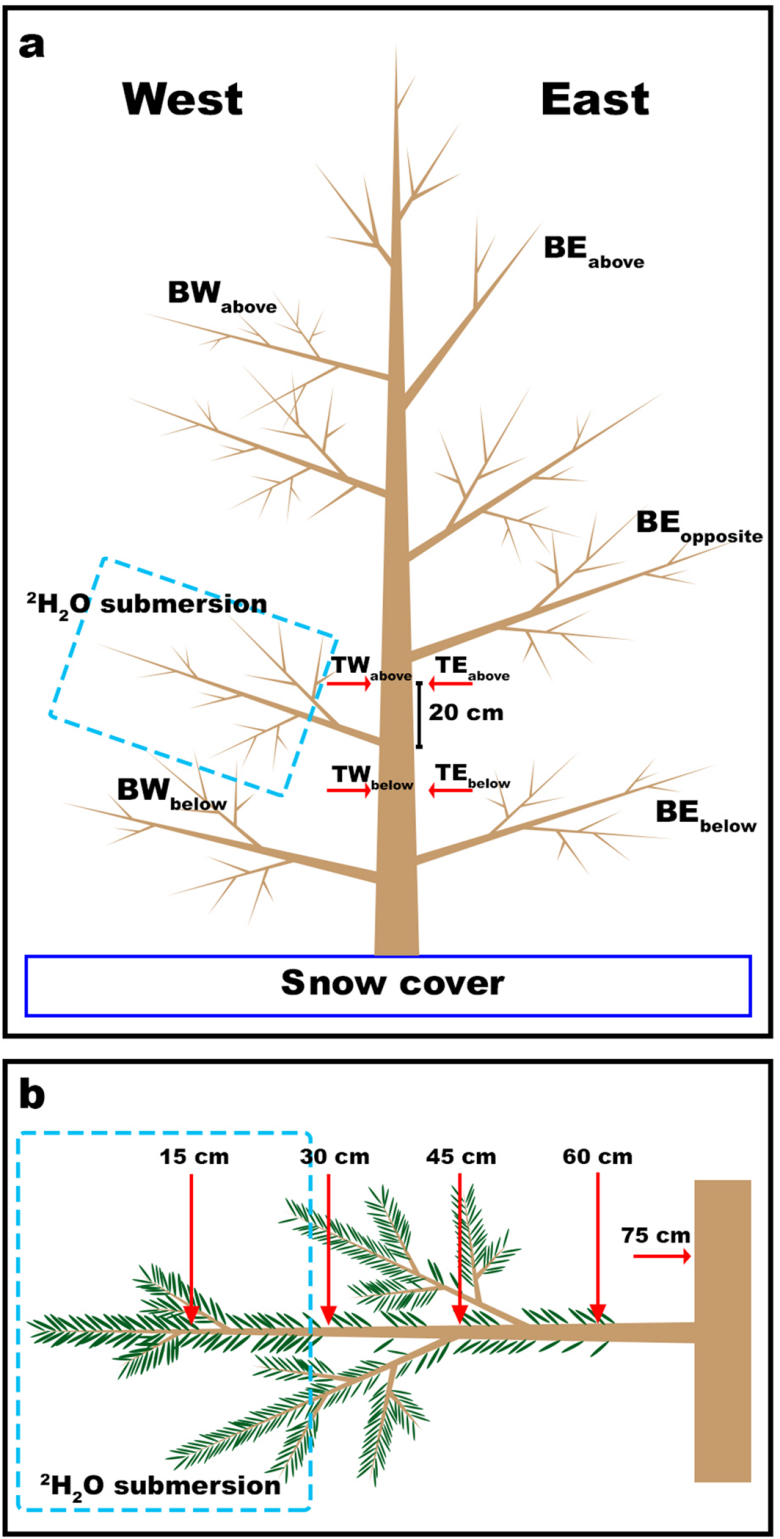
Experiment setups. (**a**) Setup used for the main experiment during the seasonal course (see “Seasonal course” in Methods). The image indicates the position of the bag filled with δ^2^H-labelled water (i.e., west at breast height) and the positions from which samples were collected. Trunk microcores (T) were named according to their position: 20 cm above and below the junction between labelled branch and trunk both on the west (W) and east (E) side of the trunk (i.e., TW_above_, TW_below_, TE_above_ and TE_below_). End-twigs were named according to the position of the sampling branch (B): west (W) or east (E) exposed, and above (BW_above_ and BE_above_), opposite (BW_opposite_ and BE_opposite_) and below (BW_below_ and BE_below_) compared to the position of the bag. (**b**) Setup used to test the branch water uptake in *P. abies* (see “Branch water uptake” in Methods). The dashed blue box indicates the position of the bag filled with labelled water. Red arrows indicate the sampling point at which microcores were extracted after bag removal (i.e.,15, 30, 45, 60 and 75 cm from the tip of the branch).

In *L. decidua*, trunk microcores (TW and TE in Fig. 1) exhibited a pronounced seasonal variation in δ^2^H (Fig. 3) and δ^18^O (Fig. 4) of the extracted water. δ^2^H of TW_above_ and TW_below_ was high in March, and a second, even higher peak was observed at the beginning of May (significant difference to controls; *P* = 0.014; see Fig. 3). At the same time, δ^2^H of TE_above_ and TE_below_ decreased. No clear course was observed in *P. abies*. In both species, highest δ^18^O were observed in March, and an increase was observed at the end of May (Fig. 4). δ^2^H and δ^18^O did not correlate with daily mean temperatures, except for δ^18^O at two trunk positions in *P. abies* (TW_below_ *P* = 0.0253; TE_above_ *P* = 0.02898). Variation in δ^18^O (and δ^2^H in the controls) reflected purely changes in natural abundance, which is driven by the balance in transpirational water loss and unlabeled water uptake.

**Figure 2:**
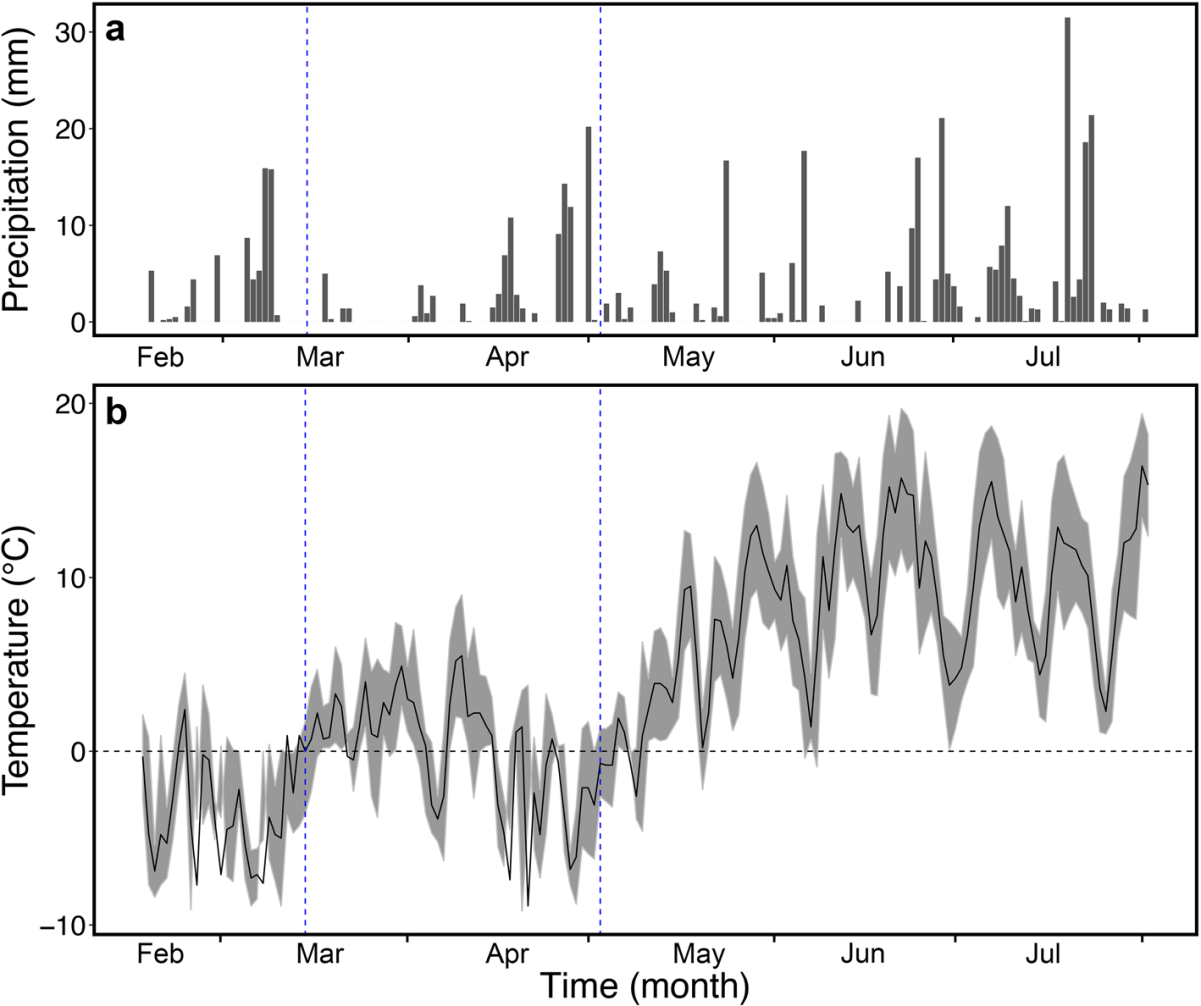
Meteorological data. Daily mean air temperature (**a**; gray area delimits daily min and max temperature) and daily cumulated precipitation (**b**) from February 16th to August 2nd, 2017, collected from a climate station near the study site (Mount Patscherkofel, 2252 m a.s.l.; provided by ZAMG Zentralanstalt für Meteorologie und Geodynamik, Austria). Blue dashed vertical lines indicate the time of bag installation (14.03.2017) and removal (02.05.2017).

**Figure 3:**
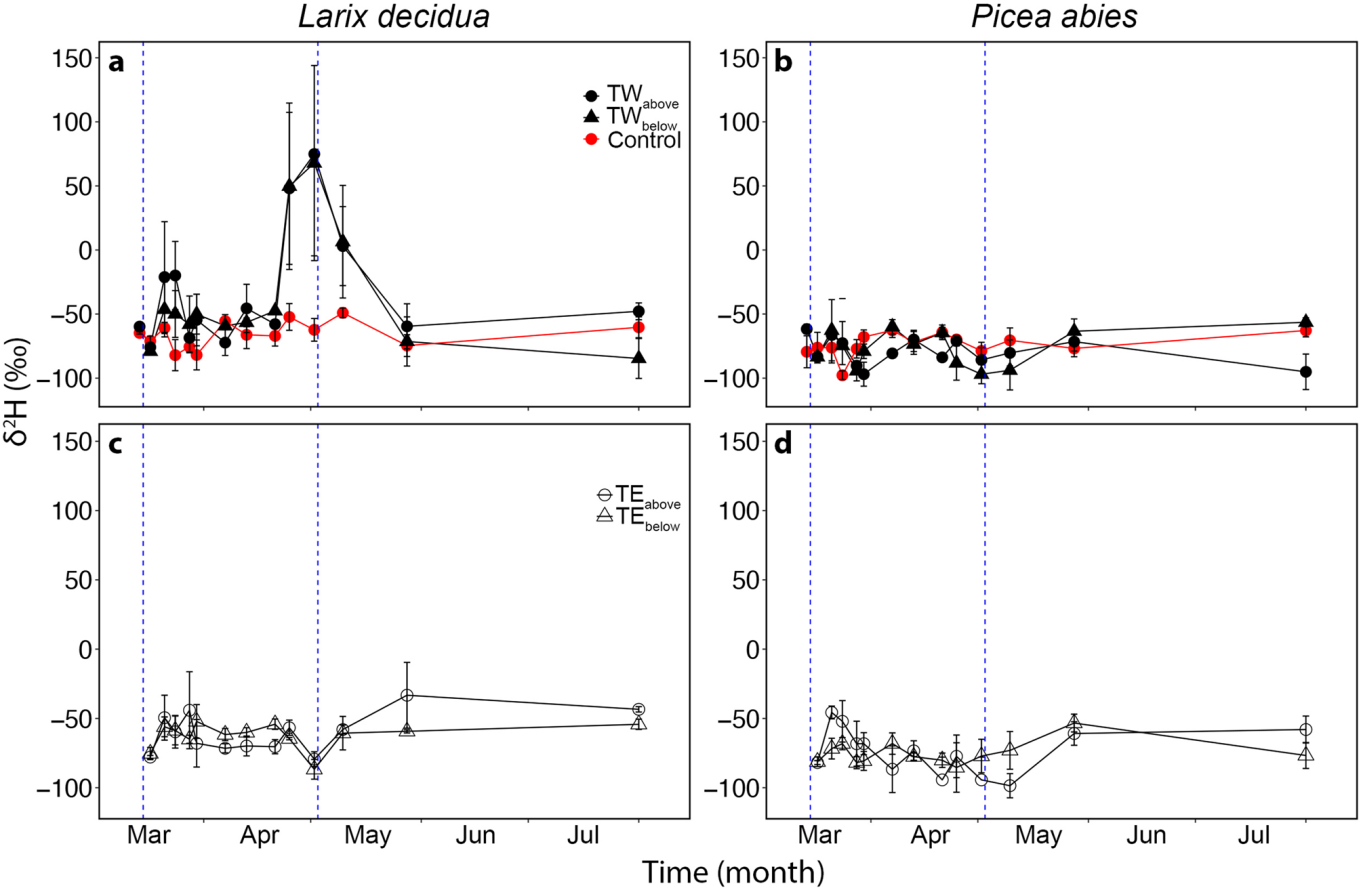
Microcores δ^2^H analyses. δ^2^H analyses (‰) of microcores extracted from the trunk of three *L. decidua* and three *P. abies* specimens growing at the alpine treeline during the seasonal course (from 14.03.2017 to 01.08.2017). Panels (**a**) and (**b**) show values from unlabeled specimens (red symbols; breast height and west exposed) and cores collected from the west side of the trunk, 20 cm above (TW_above_; full dots) and below (TW_below_; full triangles) the labelled branch (see Fig. 1 for sample nomenclature). Panels (**c**) and (**d**) show values from cores collected from the east side of the trunk, 20 cm above (TE_above_; open dots) and below (TE_below_; open triangles) the labelled branch (see Fig. 1 for sample nomenclature). Mean ± SE. Blue dashed vertical lines indicate the time of bag installation (14.03.2017) and removal (02.05.2017).

**Figure 4:**
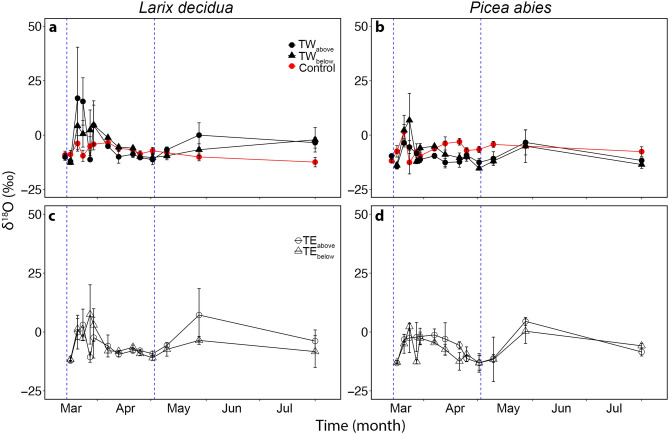
Microcores δ^18^O analyses. δ^18^O analyses (‰) of microcores extracted from the trunk of three *L. decidua* and three *P. abies* specimens growing at the alpine treeline during the seasonal course (from 14.03.2017 to 01.08.2017). Panels (**a**) and (**b**) show values from unlabeled specimens (red symbols; breast height and west exposed) and cores collected from the west side of the trunk, 20 cm above (TW_above_; full dots) and below (TW_below_; full triangles) the labelled branch (see Fig. 1a for sample nomenclature). Panels (**c**) and (**d**) show values from cores collected from the east side of the trunk, 20 cm above (TE_above_; open dots) and below (TE_below_; open triangles) the labelled branch (see Fig. 1a for sample nomenclature). Mean ± SE. Blue dashed vertical lines indicate the time of bag installation (14.03.2017) and removal (02.05.2017).

In both species, microcores collected from bagged branches (after bag removal, i.e., May 2nd) showed very high δ^2^H (*P. abies* 276.6 ± 75.8 ‰, *L. decidua* 441.3 ± 52.3 ‰), which was significantly higher than δ^2^H measured in the trunk (e.g., see Fig. 3). In contrast, δ^18^O was lower (*P. abies* -9.1 ± 1.9 ‰; *L. decidua* − 16.4 ± 4.2 ‰) than measured in trunk samples of the seasonal course (e.g., see Fig. 4).

**Figure 5:**
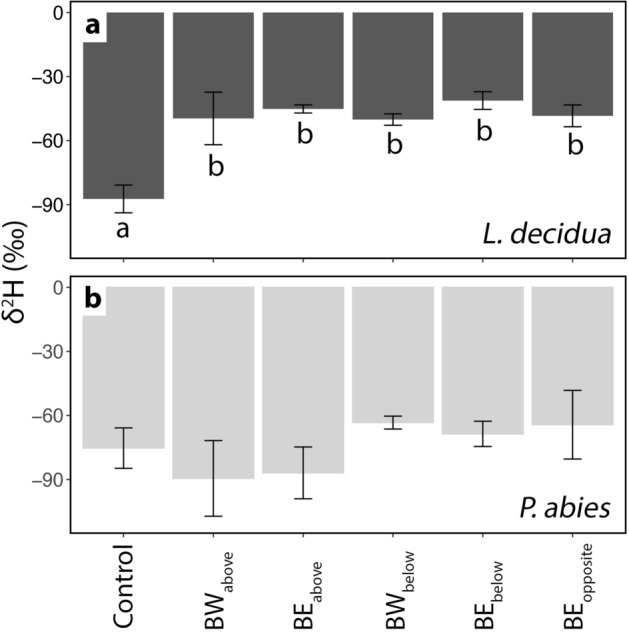
End-twigs δ^2^H analyses. δ^2^H analyses (‰) from the xylem of end-twigs (ca. 10 cm) collected at different positions within the crown of the same *L. decidua* (**a**) and *P. abies* (**b**) specimens used for cores (see Fig. 1) on April 25th, 2017. The Figure shows values from control specimens and from samples collected at different positions with respect to the labelled bag: branch above west (BW_above_) and east (BE_above_), branch below west (BW_below_) and east (BE_below_), and branch opposite east (BE_opposite_) (see also Fig. 2a for sample nomenclature). Mean ± SE. Different letters indicate significant differences (*P* < 0.05) between means. No difference was found in *P. abies*.

In end-twigs of labelled trees (BW and BE in Fig. 1), δ^2^H and δ^18^O signals were overall lower than in trunk samples, and an overall constant δ^18^O was observed in both species. A week before ending the δ^2^H-labelling treatment, in *L. decidua* (but not in *P. abies*), all end-twigs collected from labelled trees showed significantly higher δ^2^H than end-twigs collected from control trees (Fig. 5). This increase corresponded to an increase in trunk δ^2^H (see Fig. 3).

### Isotope analyses: branch water uptake

**Figure 6:**
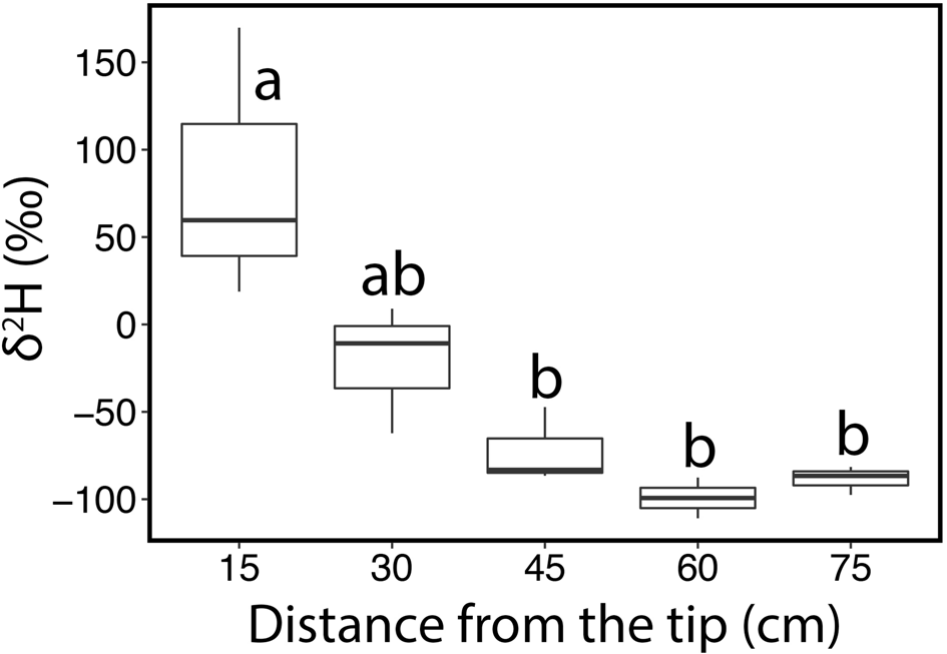
Branch water uptake. δ^2^H values (‰) measured on microcores extracted along four branches (every ca. 15 cm) of *P. abies* after 2 weeks of labelling. The point at 15 cm was submersed inside the bag, while points at 30, 45 and 60 cm were outside the bag. The point at 75 cm corresponds to the microcores collected at the trunk (see Fig. 1b). Boxplots show the median, the 25th and 75th percentiles and the whiskers show minimum and maximum values. Different letters indicate significant differences (*P* < 0.05) between means.

Samples collected along branches of *P. abies*, which were labelled for 2 weeks, showed a strong decrease of δ^2^H signal from branch tip to base (*P* = 0.0014; Fig. 6). δ^2^H decreased from 83.8 ± 45.0 ‰, in the section inside the bag (see Fig. 2b), to − 99.2 ± 45.0 ‰, at 60 cm to the branch tip.

### PLC and water potential

**Figure 7:**
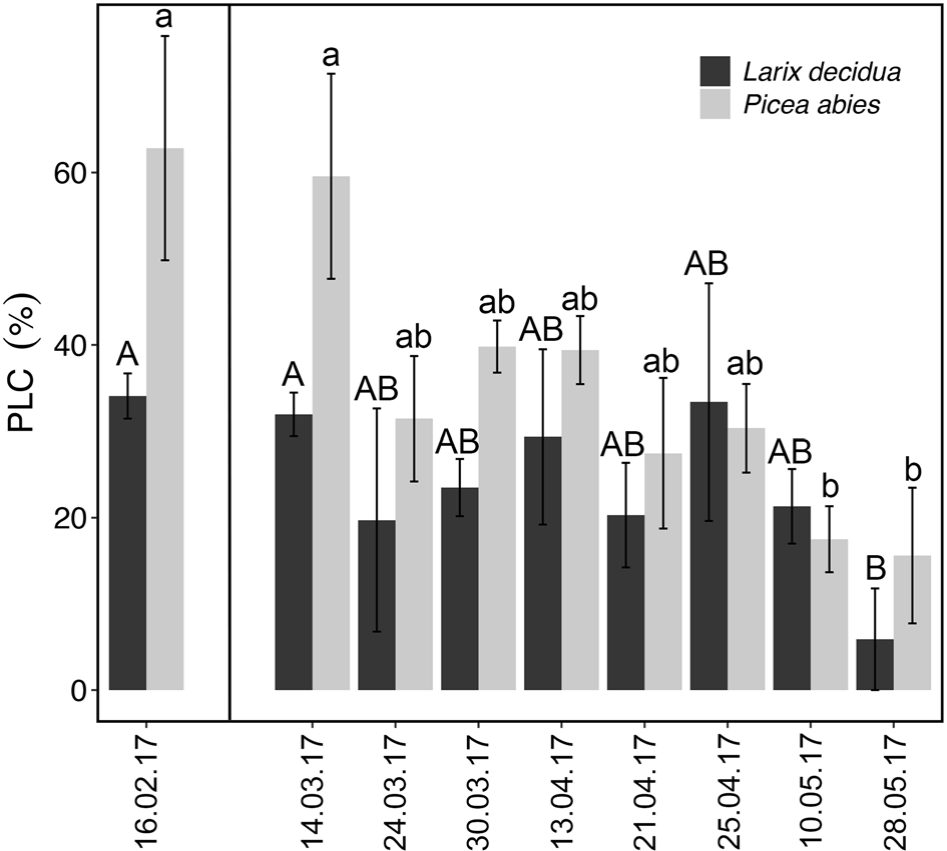
PLC measurements. Percent loss of conductivity (PLC, %) measured on branches of *L. decidua* (dark bars) and *P. abies* (gray bars) collected weekly during the seasonal course. Means ± SE. Different letters (*L. decidua*: capital; *P. abies*: lower case) indicate significant differences (*P* < 0.05) between means. The first PLC measurement (i.e., 16.02.2017) was done before the start of the labelling experiment.

In both species, percent loss of conductivity (PLC) values were higher in winter (February: *P. abies* 62.8 ± 13.0%; *L. decidua* 34.1 ± 2.6%), and progressively decreased until the end of May (*P. abies* 15.6 ± 7.8%; *L. decidua* 5.8 ± 5.9%; Fig. 7).

**Figure 8:**
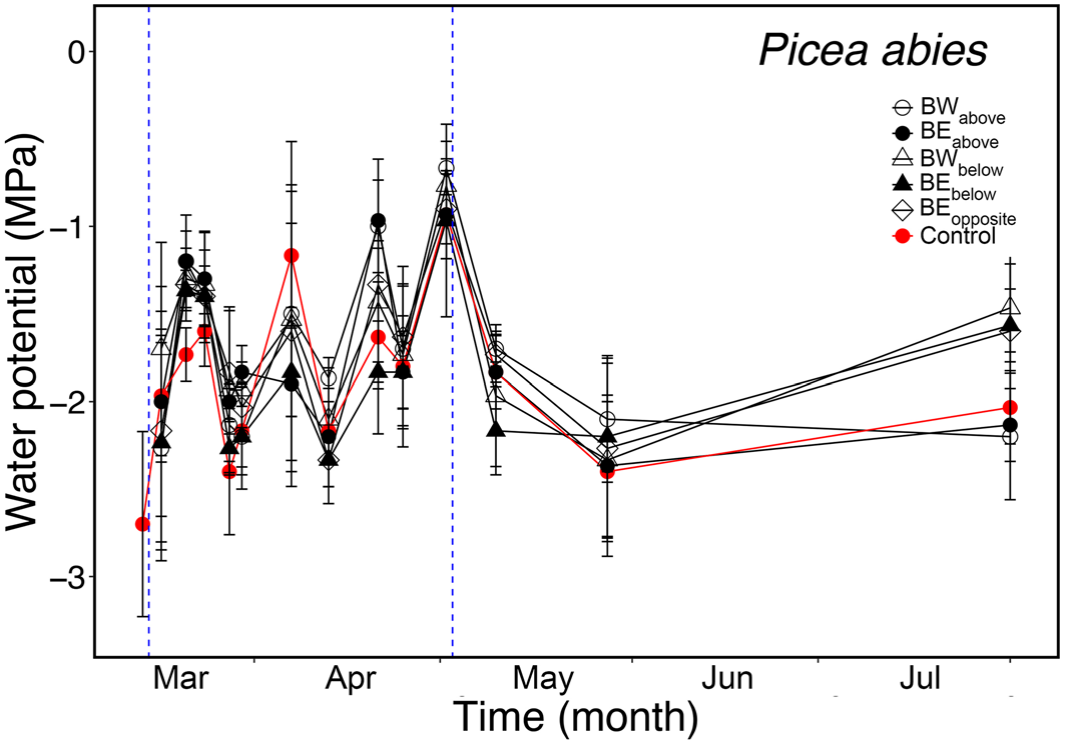
Water potential. Seasonal variations in water potential (Ψ; MPa) of *P. abies* unlabeled (red symbols; breast height and west exposed) and labelled specimens (black symbols). From labelled trees, samples were collected at different positions (see Fig. 2a): west exposed above (BW_above_; open dots) and below (BW_below_; open triangles), and east exposed above (BE_above_; full dots), below (BE_below_; full triangles) and opposite (BE_opposite_; open diamond). Mean ± SE. Blue dashed vertical lines indicate the time of bag installation (14.03.2017) and removal (02.05.2017).

In *P. abies*, Ψ showed some variation throughout the winter season and minimum values down to -3 MPa (Fig. 8). No significant difference was found between control and labelled trees. The course in Ψ did not correspond to seasonal changes in δ^18^O and δ^2^H measured on *P. abies* trunk microcores (see Figs. 3, 4).

## Discussion

Both conifer species under study were demonstrated to take up water over the branch surface and to redistribute absorbed water over significant distances within the crown. This confirms previous studies suggesting branch/foliar water uptake in conifers^3,23,26–28^ and proves for the first time that water can be redistributed over large distances from branches towards the trunk and side branches under winter conditions. However, our study shows that the extent of water absorption and redistribution is species-specific.

In *L. decidua*, increasing δ^2^H, which was observed in trunk xylem oriented to the labelled branch in March and April (Fig. 3a), clearly indicated water uptake over the branch and redistribution towards the main axis. On April 25, the labelled water was detected in all harvested end twigs (Fig. 2a), demonstrating that water had also been distributed towards neighboring branches (Fig. 5a). Labelled water was thus shifted more than 50 cm from the bag towards the trunk, where the δ^2^H signal could be found over a length of 150 ± 17 cm. The maximum observed distance of labelled samples within the axis system (i.e., distance between end twig BE_above_ and BE_below_) was 425 ± 5 cm (tree height was 330 ± 40 cm). This is surprising as temperatures were still low and ice blockages in the xylem (e.g., in shaded stem sections) may limit axial flows. The hydraulic connection, even between the most distant sections under study, must have been established at least during some of the warmer winter days. In contrast, δ^2^H values measured in *P. abies* specimens showed some variation but no clear change in trunk (Fig. 3b,d) or end-twigs (Fig. 5b) during the studied season. In accordance with findings in Mayr et al. (2014), *P. abies* specimens were obviously able to take up water through the branches, as the δ^2^H signal was detected along the main axes of labelled branches (Fig. 6). However, the δ^2^H signal ceased at about 45 cm distance from the labelled water source and thus redistribution of water did not supply the trunk with water taken up. We suggest that the evergreen *P. abies* took up water not only from the mounted bag but also from melting snow present on other parts of the tree, which reduced the relative amount of labelled water within the axis system. According to weather data, precipitation fell during cold periods, and the snow which had accumulated on the branches melted during the following warmer days (Fig. 2). This created a beneficial situation for *P. abies* trees, as water could be absorbed at different parts of the crown, and water uptake probably masked the δ^2^H signal by reducing the concentration of absorbed labelled water (see Figs. 3b, d, 5b). This effect was not relevant in *L. decidua*, which showed clear signals of the^2^H-label in the trunk. Due to its deciduous habitus, snow hardly accumulates on branches of *L. decidua*. Thus, less snow melted on branches, which led to a smaller dilution of the label compared to *P. abies*. The ability of long-distance water transport within trees might become crucial when external water is not equally available across the crown (e.g., in case of inhomogeneous snow cover). Water absorbed in one crown part can potentially be transported to other crown sections, which may be particularly important for treeline conifers exhibiting complex within-tree patterns of both water potential and embolism under winter conditions^4^.

Trunk δ^18^O values in water showed an overall increase towards the end of March in both species and was more pronounced in *L. decidua* (Fig. 4). This change corresponded to an increase in temperatures (Fig. 2a), which likely caused thawing of frozen xylem sections, enabling a redistribution of water and equilibration of Ψ within the crown. Another increase in trunk δ^18^O occurred at the end of May in both species when temperatures raised again (Fig. 2a). At this time, access to soil water was reestablished and the recovery in PLC occurred (Fig. 7). Re-activation of soil water uptake caused changes in the δ^18^O signal due to the different signature in soil water.

Previous studies on conifers supposed both needles^23,26–28^ and bark^24^ to be responsible for water uptake. Katz et al.^25^ specifically found pathways for the absorption of water and solutes in *P. abies* bark and ray tissue, while Mayr et al.^3^ suggested partly opened stomata^33^ and high cuticular conductance of *P. abies* needles^1,34^ to provide possible points of water uptake from melting snow. Moreover, two previous studies^3,6^ reported an increase in the amount of aquaporins in the needle endodermis and in phloem cells during the recovery in springtime. Aquaporins might be important for direct water absorption and shifts, as they reduce resistances in symplastic pathways ^20,35,36^. Mayr et al.^3^ also observed starch accumulation in phloem and needle tissues, which might provide the required driving force for local changes in Ψ^37^ by being degraded into osmotically active sugars and released into embolized conduits to create a driving force for water inflow^38^. Our study did not focus on the mechanism of water uptake, but the labelling experiment on the deciduous *L. decidua* clearly indicated that water absorption also occurred via the bark. Earles et al.^24^ suggested water to follow symplastic pathways for entering the xylem over the bark and absorbed water to enable repair of xylem embolism in *Sequoia sempervirens*. Water uptake at the branch level of *P. abies* might occur by a combination of both bark and foliar absorption.

In accordance with previous studies^1,3,5,6^, harsh winter conditions at high elevation induced high embolism rates (PLC: *P. abies* 62.8 ± 13.0%, *L. decidua* 34.1 ± 2.6%; Fig. 7). *L. decidua* showed lower PLC than *P. abies* most likely due to the lack of needles, which reduced winter transpiration ^1,10,39,40^. We expect Ψ of *L. decidua* (which could not be measured; see methods) thus to have been less negative than in *P. abies*, which reached ca. -3 MPa at a minimum (Fig. 8). Hydraulic conductivity started to recover during springtime (March–April; Fig. 7), when the snow cover was still present and soil water was not available. This early recovery might thus be supported by absorbed water. In May, a further recovery (PLC: *P. abies* 15.6 ± 7.8%, *L. decidua* 5.8 ± 5.9%; Fig. 7) occurred, corresponding to the increase in temperature (Fig. 1a). In the labelling experiment, δ^2^H signals indicated redistributed water in *L. decidua* in late March and early May (Fig. 3), which corresponds well with the observed two phases of xylem recovery. Long-distance transport of water absorbed via branches during late winter and early spring days with favorable weather conditions might thus be crucial for achieving a balanced water status. It may enable the transport of water to parts of the plant, where local water absorption is not possible, and support the repair of embolized conduits. Previous studies^6^ indicated that past-winter recovery can completely counterbalance winter embolism, though there might be legacy effects (e.g. due to cavitation fatigue or reductions in the conductive area)^41,42^.

It is unclear how climate change might affect water absorption and redistribution in treeline conifers. Predicted increase in temperature is expected to shift alpine treelines to higher elevation as the limit of mountain trees is determined by temperatures^43,44^, though other factors can modulate distributional limits at small scale^45–50^. Accordingly, improved tree growth and recruitment was already observed at several different treelines across the globe^45,51–54^. Higher temperatures during spring may support metabolic processes involved in the recovery from winter drought, but they may also lead to a reduction of winterly snow cover^55,56^. A lack of snow protection will increase frost drought and freeze–thaw stress during winter (unless temperatures are high enough to enable soil thawing) and reduce the amount of melting snow on crowns, limiting water recourses for branch water uptake. Thus, formation and recovery of winter embolism in treeline conifers will not only be influenced by warmer temperatures but also by changes in (local) precipitations patterns and dynamics. Changes may be especially pronounced in late winter and spring (as well as autumn) when small temperature increases are sufficient to cause precipitation changing from snowfall to rain.

The present study suggests that not only the evergreen *P. abies*^3,6^, but also the deciduous *L. decidua* is able to take up water over branches during late winter and spring, indicating that this may potentially be a more widespread ability of treeline conifers in general. It also demonstrates that absorbed water can be redistributed over long distances within the crown, which is an important prerequisite for embolism repair throughout the axes system of treeline conifers. This redistribution can occur over meters within branches and the trunk, which is impressive considering the still winterly conditions, with frozen soil and frequent subzero temperatures during nights and respective ice blockages in xylem sections. It is thus likely that melting snow is an important source for branch water uptake, and results on the deciduous species *L. decidua* clearly indicated bark to participate in the process of water absorption. Absorbed and redistributed water may contribute to the repair of winter xylem embolism as well as restore water reservoirs prior to the vegetation period. Though, in light of climate change, this specialized mechanism might be compromised by a reduction in snowfalls, thus exposing trees to harsher winter conditions and impaired hydraulics. We encourage further studies investigating water absorption and redistribution in additional alpine treeline species on both bark and leaf level to better understand the physiological mechanisms behind these processes.

## Methods

### Plant material

This study was performed on *Larix decidua* Mill. and *Picea abies* (L.) H. Karst. trees growing at the alpine treeline (2150 m a.s.l.). The study site was located on a south-west exposed slope on Mount Patscherkofel in Tyrol, Austria (47°12′ N, 11°27′ E). The site was accessed by use of touring skis, which enabled measurements in a widely undisturbed area but limited the number of experiments and replicates. Species identification was carried by the main author (Adriano Losso), and permission for sampling was granted by the local authorities. Single standing trees (330 ± 40 cm tall) within an open stand in a mixed forest were chosen for measurements. Six trees per species (3 controls and 3 for the experiment) were selected for the labelling experiment during the seasonal course (see below) and four *P. abies* trees were selected for the branch water uptake experiment (see below). For percent loss of conductivity measurements (PLC), 1-m-long branches were collected from four additional control trees at each time interval (see below “Seasonal course”). No voucher specimen of the plant material under study was collected and deposited in a publicly available herbarium. All measurements were carried out in accordance with institutional, national, and international guidelines and legislation.

### Seasonal course

For this experiment, samples were collected from winter to summer 2017, with higher sampling frequency during the transition from late winter to spring (March–May 2017). The first PLC measurements were performed on February 16 (i.e., mid-winter) to check the overall water status of trees under study, whereas the labelling experiment began on March 14 and sampling was roughly done twice a week until May 28 (see also below). One additional sampling campaign was conducted on August 1 to monitor eventual remains of deuterium within trees.

On March 14, 2017, one west exposed branch (at breast height) per tree was bagged in situ. West exposure was favored over other exposures due to facilitated working conditions as well as to allow labelled branches to experience daily temperature variation without being compromised by prolonged shadow (i.e., frozen labelled water). However, branches were partly shaded by higher crown parts so that radiational overheating was avoided. Temperatures inside and outside the bags were monitored (Minikin datalogger, EMS, Brno, Czech Republic) on two trees per species until bag removal. Bags (transparent, 4 L volume) were filled with about 1–1.5 L of δ^2^H-labelled water (100 ppm ^2^H_2_O [v/v]). On the same day, prior to bag installation, one end-twig (ca. 10 cm) from the selected branch was harvested, and one microcore was extracted at breast height from the west exposed side of the trunk. From March 17 to May 2, 2017, the study site was visited twice a week, and four trunk microcores and five end-twigs were collected at different positions of each tree. A scheme of sampling positions (named with respect to the bagged branch: above, below or opposite) is given in Fig. 1. To enable repeated sampling at the same position, microcores were extracted either a few millimeters below or sidewards of the previous measurement.

Trunk microcores were 2 mm in diameter and 8–10 mm long cylindrical xylem samples collected using a 35-mm-long Trephor microcorer^57^. Microcores were immediately secured in aluminum strips and inserted in 4 mL glass vials, while intact end-twigs (ca. 10 cm and with needles) were placed in sealed plastic bags. Samples were then transported to the laboratory and frozen until isotopic analysis.

On May 2, 2017, bags were removed and from each branch, a microcore was collected from the main axis section previously immersed in water. Three additional sampling campaigns, following the sampling protocol given above, were done on May 10 and 28, and August 1, 2017, to monitor eventual remains of deuterium within trees.

Three additional trees per species were used as control trees: on each sampling date, one microcore and one end-twig per tree were sampled at breast height (both west exposed), transported to the laboratory and frozen until isotopic analysis.

For a monitoring of native hydraulic recovery, on February 16, and from March 14 to May 28, 2017, one additional west-exposed 1-m-long branch was collected about every 10 days from each control tree, wrapped in black plastic bags and transported to the laboratory for hydraulic measurements (please see below “PLC and water potential”). These measurements were thus conducted when hydraulic recovery was expected to occur^3,6^. PLC measurements on bagged branches were not possible due to the destructive nature of these measurements.

At each sampling date of the seasonal course (see above), additional end-twigs were collected from *P. abies* specimens (both labelled and control) for water potential (Ψ) measurements (please see below “PLC and water potential”). Due to the deciduous habit of *L. decidua*, Ψ measurements were not possible during winter.

### Branch water uptake measurements

In addition to measurements over the winter season, an experiment to assess the amount of water absorbed from a single branch was performed: On March 14, 2017, four additional *P. abies* specimens were selected and a west exposed branch (ca. 75 cm long) at breast height was bagged in situ. Bags (transparent, 4 L volume) were filled with about 1–1.5 L of δ^2^H-labelled water (100 ppm ^2^H_2_O [v/v]). After 2 weeks, bags were removed and four microcores were collected along each branch (15, 30, 45 and 60 cm distance from the branch tip, respectively; Fig. 1b). One additional microcore was collected from the trunk about 5 cm above the insertion point of the bagged branch. Microcores were individually secured in aluminum strips, inserted in 4 mL glass vials, transported to the laboratory and frozen until isotopic analysis.

### Isotope analyses

An induction module (IM) combined to a cavity ringdown spectroscopy (CRDS) analyzer (both Picarro Inc., Sunnyvale, CA, USA) allowed the determination of water isotopes abundance (δ^18^O and δ^2^H) in organic material^58–61^. Microcores in vials were slowly defrosted and then analyzed one by one with the IM. For each end-twig, a segment (diameter 2 mm, length 8–10 mm) was cut, debarked, and secured in aluminum strips inside a 4 mL sealed glass vial. Samples were then slowly defrosted and analyzed one by one with the IM. The IM inductively heats up the aluminum strips within each individual vial for several minutes (the IM offers a setting named ‘wood’, which heats up the wood samples for a cycle of 360 s). The close contact between the wood sample and the heated aluminum strip vaporizes any water contained inside the sample and allows the extraction of water from the samples. The IM is equipped with a micro-combustion module, which reduces interferences related to the presence of organic compounds^62^ potentially released while plant samples are heated^63^.

Control measurements proved that three measurement cycles of standard solution (δ^18^O–16.6 ‰; δ^2^H–100.4‰) were enough to eliminate any memory effect (data not shown) of δ^2^H-labelled water (100 ppm ^2^H_2_O [v/v]). Test measurements also proved that a wood volume of 7–12 mm^3^ was estimated for successful extraction of 3–5 μL of water from each sample and control measurements indicated a complete water extraction without samples carbonization (data not shown). Variation in Ψ did not influence extracted water and δ^2^H in branches (data not shown).

### PLC and water potential

Percent loss of conductivity (PLC) was quantified with a modified Sperry apparatus^64,65^ on three to four sections of the 1-m-long branch axis (ca. 4–5 cm-long) and averaged per branch. Each section was cut under water, the bark was removed, and samples were trimmed several times with a sharp carving knife to gradually release tension and remove micro-bubbles^66,67^, and connected to the apparatus. PLC was then determined by measuring the hydraulic conductivity before and after removal of xylem embolism by repeated high-pressure flushes^64^. Conductivity measurements (4 kPa) and flushing (80 kPa) were done with distilled, filtered (0.22 mm) and degassed water containing 0.005% (v/v) Micropur (Katadyn Products) to prevent microbial growth. Flushing was repeated until measurements showed no further increase in conductivity. All hydraulic measurements were conducted at room temperature (ca. 21–22 °C). Conductivity values were corrected for water viscosity at 20 °C, and PLC was calculated as PLC = (1 − *K*_min_/*K*_max_) * 100, where *K*_min_ is the initial conductivity and *K*_max_ is the maximal conductivity.

For *P. abies*, Ψ of end-twigs was measured with a Scholander apparatus (model 1000; PMS Instrument Company, Corvallis, OR, USA). In labelled-trees, end-twigs were collected from each branch used for isotopic analysis (i.e., BW_above_, BW_below_, BE_above_, BE_opposite_ and BE_below_; see Fig. 1) and Ψ of branches at the same position were averaged. In controls, one end-twig per tree was collected at breast height, and values were averaged.

### Meteorological data

Meteorological data from a climate station near the study site (Mount Patscherkofel, 2252 m a.s.l., provided by ZAMG Zentralanstalt für Meteorologie und Geodynamik, Austria) were used to calculate daily cumulated precipitation and minimum, maximum and mean air temperature during the seasonal course (from February 16th to August 1st, 2017; Fig. 2).

### Statistics

All values are given as mean ± standard error. Differences were tested using one-way ANOVA followed by Tukey’s post hoc comparison (PLC, seasonal course of microcores and end-twigs δ^18^O and δ^2^H, Branch water uptake experiment),﻿ after testing for normal distribution and homoscedasticity, while correlation analysis was carried out using the Pearson product–moment correlation (Ψ, δ^18^O and δ^2^H versus temperatures). All tests were performed in R v. 3.6.2 (R Development Core Team, 2017) at a probability level of 5%.
